# In Vitro Antibacterial Potential of Herbal Beverage Extracts From Cinnamon, Clove, and Thyme and Their Interactive Antimicrobial Profile With Selected Antibiotics Against Drug-Resistant Clinical Pathogens

**DOI:** 10.1155/jotm/9916282

**Published:** 2025-05-20

**Authors:** Armel Jackson Seukep, Ojong Carlos Gerard Ojong, Helene Gueaba Mbuntcha, Valaire Yemene Matieta, Elisabeth Menkem Zeuko'o, Arnaud Fondjo Kouam, Victor Kuete, Lucy Ayamba Ndip

**Affiliations:** ^1^Department of Biomedical Sciences, University of Buea, Buea, Cameroon; ^2^Department of Biochemistry, University of Dschang, Dschang, Cameroon; ^3^Department of Microbiology and Parasitology, University of Buea, Buea, Cameroon; ^4^Laboratory for Emerging Infectious Diseases, University of Buea, Buea, Cameroon

**Keywords:** antibacterial activity, cinnamon, clove, combination testing, herbal beverages, phytochemical analysis, thyme

## Abstract

The increase in antibiotic resistance has increased the demand for new and safe therapeutic options. Herbal beverages, whether used alone or combined with standard antibiotics, have shown promise in combating drug-resistant bacteria. This study investigated the antibacterial activity and combinatorial efficacy of common herbal beverages prepared from clove, cinnamon, and thyme. The inhibitory and cidal effects were examined using MIC and MBC on a panel of 14 multidrug-resistant strains and clinical isolates (resistant to ciprofloxacin (CIP), tetracycline (TET), and erythromycin (ERY)), including *Staphylococcus aureus*, *Salmonella* and *Shigella* species, *Escherichia coli*, and *Pseudomonas aeruginosa*. The combinatorial efficacy was further evaluated using a fractional inhibitory concentration index (FICi). Qualitative phytochemical screening of the plant extracts followed established protocols. The tested botanicals showed inhibitory effects against all 14 tested bacteria, with varying degrees of potency (MICs ranged from 13.33 ± 2.67 to 1024 ± 0.00 μg/mL). The aqueous and hydroethanolic extracts of clove demonstrated the highest activity, with most MIC values ranging from 13.33 ± 2.67 to 256 ± 0.00 μg/mL, indicating excellent to good efficacy. When combined with TET, CIP, and ERY, clove extracts exhibited significant synergistic and additive interactions, leading to more than a 100-fold reduction in the MICs of the antibiotics in some cases. The most notable synergistic interactions were observed with the combination of clove hydroethanol extract with TET (FICi = 0.078 ± 0.016) against *P. aeruginosa*. The findings indicate possible optimization of antibiotic treatment strategies using these combinations, which may help mitigate antibiotic resistance and improve patient outcomes. However, an antagonistic effect was observed with the clove aqueous extract and CIP on *S. aureus*, which may require further evaluation. Phytochemical analysis revealed the presence of several major bioactive secondary metabolites, including phenols, flavonoids, tannins, anthocyanins, saponins, and alkaloids. Overall, the tested botanicals, particularly clove, demonstrate considerable potential in fighting drug-resistant bacteria, either through direct action or by enhancing the effectiveness of existing antibiotics. Further, in vivo testing and investigation of the mechanisms behind the active combinations are recommended to assess their overall efficacy.

## 1. Introduction

Antibiotic resistance has become a major concern globally, posing a significant threat to public health. In recent years, there has been a significant increase in the emergence of antibiotic-resistant bacteria, making conventional antibiotics ineffective for treating infectious diseases [1]. The bacterial species examined in this study include *Staphylococcus aureus*, *Pseudomonas aeruginosa*, *Escherichia coli*, *Salmonella* species, and *Shigella* species. These bacteria are clinically relevant and are often linked to a variety of infections and diseases. They are associated with conditions such as wound infections, bacteremia, sepsis, food poisoning, and diarrheal illnesses. Some of these bacteria, particularly *S. aureus* and *P. aeruginosa*, are major contributors to healthcare-associated infections and present a significant challenge due to their resistance to antibiotics. These bacteria are known for their high levels of resistance to multiple classes of antibiotics, which leads to increased rates of morbidity and mortality among the affected individuals [1–3]. They rank among the high-priority pathogens that guide research and the discovery of innovative antibacterial therapeutics, as reported by the World Health Organization (WHO) [2]. Finding new strategies or alternatives to combat antibiotic resistance in these bacteria is crucial. Current challenges in addressing antibiotic resistance include the lack of new antibiotic development [4]. Moreover, the high cost and limited availability of existing antibiotics in certain regions present significant barriers to effective treatment strategies [5].

There is growing interest among scientists and researchers in exploring alternative sources for antibiotics, such as plant-derived products, including essential oils, extracts, and herbal teas. The herbal teas are traditionally used, and recent studies suggest their antimicrobial properties [6, 7]. These alternatives have shown promise in helping to overcome the limitations and challenges associated with conventional antibiotics [6]. Medicinal plants have long been recognized for their therapeutic properties and are used in traditional medicine due to their rich content of natural compounds, such as polyphenols, flavonoids, alkaloids, terpenoids, and essential oils, all of which exhibit antimicrobial activities [7]. Furthermore, studies have demonstrated that the antibacterial properties of dietary plants and herbal teas, whether used alone or in combination with standard antibiotics, could help mitigate and overcome resistance mechanisms by inhibiting specific resistance tactics expressed by them [8–20]. Antimicrobial agents are among the most commonly prescribed groups of drugs, highlighting the potential for concurrent use with popular herbal beverages [20]. In Cameroon, a cross-sectional survey of adults aged 18–65 revealed a high consumption of herbal teas (89.3%). Most participants reported using these teas for the prevention and treatment of COVID-19 (67.9%), malaria (59.7%), and typhoid fever (35%). The survey also indicated a generally positive opinion among participants and a willingness to use these teas if prescribed in healthcare settings [21]. Herbal teas therefore could serve as a highly effective alternative treatment and a supportive addition to traditional medications. Besides their beverage properties, herbal teas are employed worldwide for treating various human diseases, including bacterial infections. Research on herbal teas and their components is still limited [18]. However, some studies have demonstrated their impressive abilities to combat various diseases, including drug-resistant bacterial infections [22]. Hacioglu et al. [18] examined the antimicrobial activities of 31 aqueous tea infusions, both individually and in combination with antibiotics or antifungals, against standard and clinical isolates. The herbal extracts were effective against most of the microorganisms studied. In the combination experiments, different effects, such as synergistic, additive, and antagonistic interactions, were observed between the teas and the antimicrobials. Notably, synergy was more frequently found between ampicillin, ampicillin-sulbactam, or nystatin and the various tea combinations [18]. Another study by Venkatesan et al. [23] highlighted the promising antibacterial and antifungal properties of *Hibiscus sabdariffa* L. (rosella), a plant commonly used for making hot or cold beverages. Additionally, research exploring the synergistic effects of *Moringa oleifera* extracts with antibiotics showed a significant reduction in the minimum inhibitory concentration (MIC) of the antibiotics when used in combination with *M. oleifera* against bacteria such as *Klebsiella pneumoniae*, *E. coli*, and *S. aureus*, along with considerable public acceptance of this decoction for improving health [24]. A study by Alkufeidy et al. [25] assessed the antimicrobial properties of green tea extract and the derived phytochemicals including catechin and benzoyl peroxide, and their combination with antibiotics against acne-causing bacteria, including *Staphylococcus epidermidis*, *S. aureus*, and *Propionibacterium acnes*, isolated from the skin of clinical subjects. Both aqueous and solvent extracts of green tea demonstrated antibacterial activity against the screened bacteria, which was further enhanced when combined with existing drugs. Overall, reviews have emphasized the nutraceutical potential of herbal beverages in managing various pathologies, including infectious diseases [26]. The findings regarding aqueous herbal teas and solvent extracts underscore a promising approach to discovering effective agents against both susceptible and drug-resistant bacteria. Most importantly, these preparations are gaining significant acceptance among the population. Our study focused on three plants (clove, cinnamon, and thyme) that are commonly used in Cameroon to prepare herbal teas.

Clove (*Syzygium aromaticum*, Myrtaceae) is a fragrant spice that originates from Southeast Asia. It has a long history of use in traditional medicine for treating toothaches, wounds, digestive issues, inflammation, and respiratory infections [27]. Thyme (*Thymus vulgaris*, Lamiaceae), a bushy herb native to the Mediterranean, is commonly used to address respiratory problems, promote wound healing, alleviate diarrhea and stomach aches, manage arthritis, soothe sore throats, and reduce hypertension [28]. Cinnamon (*Cinnamomum cassia*, Lauraceae), which originated in China, is utilized in folk medicine to treat conditions such as coughs, diarrhea, pain, respiratory disorders, and gynecological issues [27]. These traditional uses suggest that these plants may possess antimicrobial properties [18–28], making them promising candidates for research into their effectiveness against antibiotic-resistant bacteria. Recent scientific investigations have begun to explore the mechanisms behind these traditional practices, particularly focusing on their essential oils. For instance, clove essential oil has shown antibacterial activity against various pathogens, a property attributed to eugenol, its major component, which disrupts bacterial cell membranes [27, 29]. Similarly, thyme essential oil contains thymol and carvacrol, both of which exhibit antibacterial properties by potentially interfering with cellular respiration and protein synthesis [30]. Cinnamon, recognized for its distinctive aroma due to cinnamaldehyde, has also demonstrated antibacterial activity, possibly by damaging bacterial cell walls and inhibiting the production of virulence factors [31]. Furthermore, research has indicated that these herbal remedies can enhance the effectiveness of traditional antibiotics through synergistic interactions, which can lead to increased antibacterial activity, resistance modification, and potentially even the re-sensitization of bacteria to antibiotics [7, 32]. Additional established benefits of these plants include antioxidant, antidiabetic, anti-inflammatory, antifungal, antimalarial, and anti-proliferative activities [33]. These selected plants are not only used as spices but are also commonly consumed as tea in many cultures, particularly in Cameroon, to help prevent or manage infectious diseases and strengthen the immune system. However, few studies focused on the antimicrobial potential and combinatorial efficacy of beverage-prepared extracts. Addressing this knowledge gap, the current study aims to investigate the potential of herbal beverages made from clove, cinnamon, and thyme, both individually and in combination with traditional antibiotics, to combat the growing threat of drug-resistant bacteria.

## 2. Materials and Methods

### 2.1. Plant Material: Collection and Authentication

Barks of *Cinnamomum cassia* (L.) D.Don (cinnamon), buds of *Syzygium aromaticum* (L.) Merr. & L.M.Perry (clove), and leaves of *Thymus vulgaris* L. (thyme) were sourced from a local market in Douala, Littoral Region of Cameroon, in January 2024. Authentication of these plants was conducted at the Cameroon National Herbarium (HNC, Yaounde), comparing them to the voucher specimens numbered 22309/SRFCam, 506167/HNC, and 42851/SRFCam, respectively. The plant names were further verified using https://www.worldfloraonline.org, accessed on January 20, 2024. Careful preparation of the samples included thorough rinsing in clean running tap water and several days of air drying before each was separately ground using the RAF Multifunctional Coffee Grinder R-7113 (RAF, Pakistan).

### 2.2. Plant Extraction Procedure

Extracts were prepared using either water or 70% ethanol as solvents. Water was chosen to simulate typical conditions for preparation before consumption, while ethanol was utilized to enhance the solubility of both polar and non-polar functional compounds found in the herbal teas. The aqueous extracts were prepared using two standard procedures: infusion for thyme leaves and decoction for cloves and cinnamon barks. These methods were selected because they are the most common and effective techniques for preparing these plants, similar to how tea is made. For the organic extracts, maceration with 70% ethanol was employed. The choice of 70% ethanol was made to achieve an optimal balance in extracting a wide range of compounds from the leaves and stem bark. This concentration provides sufficient polarity to dissolve polar molecules while also retaining the ability to extract some less polar compounds [34].

#### 2.2.1. Infusion

An aqueous tea infusion was prepared by adding 1 L of boiling water (80°C) to 100 g of dried thyme leaves [18–20]. This mixture was allowed to steep overnight to ensure that sufficient phytochemicals were extracted into the water. After steeping, the infusion was filtered using a regular mesh filter, followed by filtration with Whatman grade 1 filter paper. This filtration process was repeated twice with the remaining residue. The resulting suspension was then distributed in small volumes onto stainless steel plates and dried in an oven at 40°C until all traces of water had evaporated. The dried extract was then carefully transferred to an airtight glass bottle and stored at 4°C until needed for testing.

#### 2.2.2. Decoction

For the clove and cinnamon extracts, the decoction method was employed. In this case, 50 g of plant powder was boiled in water for 30 min and then left to steep overnight to ensure proper extraction of the phytochemicals [18–20]. The filtration process and drying followed the same steps as described for the infusion method.

#### 2.2.3. Maceration

The hydroethanolic extracts were prepared using a standard maceration procedure with a 70% ethanol solution (1:10 w/v) [20]. Briefly, a mass (50 g of clove and cinnamon and 100 g of thyme) of dried plant material was soaked in ethanol (1:10 w/v) and left to macerate for 48 h, with constant stirring every 6 hours. Afterward, the mixture was filtered using a tea filter, followed by Whatman grade 1 filter paper. This filtration process was repeated two more times with the remaining residue. The resulting suspensions were then dried at 40°C and stored at 4°C until further use.

The extraction yield for each method was calculated based on the initial mass of the plant powder using a specified formula (1)

(1) $$\begin{array}{c} \text{Extraction yield}=\left(\frac{\text{weight of crude extract}}{\text{weight of initial air}-\text{dried powder}}\right)\times 100. \end{array}$$

### 2.3. Standard Drugs and Other Chemical Substances

Antibiotic molecules with a purity of over 95%, including tetracycline (TET), ciprofloxacin (CIP), and erythromycin (ERY), were utilized for the combination assay. These antibiotics come from various broad-spectrum classes (TET, fluoroquinolone, and macrolide, respectively) and were chosen due to their common application in treating diseases caused by the studied pathogens. Dimethyl sulfoxide (DMSO) (Pure Pharma Grade, USP) was used to solubilize the plant extracts. The final concentration of DMSO was less than 2.5% (v/v), which is reported to be innocuous to bacteria [8–17]. Ethanol (70%) served as the solvent for organic extraction. *Para*-iodonitrotetrazolium chloride (INT) was employed as an indicator for microbial growth. All chemicals were sourced from Shanghai Macklin Biochemical (Shanghai, China). Hydrochloric acid, lead acetate, sodium chloride, ferrous sulfate solution, acetic anhydride, chloroform, and potassium iodide, all of analytical grades, were used for the qualitative phytochemical analysis.

### 2.4. Screening of Major Classes of Plant Secondary Metabolites

The major plant secondary metabolite classes including tannins, saponins, phenols, flavonoids, anthocyanins, alkaloids, and terpenoids were qualitatively screened according to the phytochemical method previously reported [35]. The procedure is summarized in Table 1.

### 2.5. Microorganisms and Culture Media

The bacteria used were drug-resistant strains and clinical isolates. They included *Staphylococcus aureus* (SANR 46003 and SA RHB), *Salmonella enteritidis* (SENR 13555 and SE CPC), *Salmonella typhimurium* (STM ATCC 14028 and STM CPC), *Salmonella typhi* (ST CPC and ST RHB), *Salmonella paratyphi* B (SPT-B CPC), *Escherichia coli* (ATCC 25922 and EC RHB), *Shigella dysenteriae* (SD CPC), *Shigella flexneri* (SFNR 518), and *Pseudomonas aeruginosa* (PA RHB). They were obtained from the American Type Culture Collection (ATCC), Biodefense and Emerging Infections Research Resources Repository (BEI resources for all NR), Center Pasteur Cameroon (CPC), and the Regional Hospital of Bamenda (RHB).

Mueller-Hinton Agar (MHA) (Chaitanya Agro Biotech Pvt. Ltd, India) was used for the maintenance and culture of bacterial strains, while Mueller-Hinton Broth (MHB) (Chaitanya Agro Biotech Pvt. Ltd, India) was utilized for broth microdilution to determine the MICs and minimum bactericidal concentrations (MBCs) of the test extracts, as well as for conducting checkerboard assays to assess fractional inhibitory concentrations (FICs). The isolates and strains were maintained on an MHA agar plate at 4°C and subcultured (activated) on fresh agar plates 18–24 h before any antimicrobial testing. The culture media were prepared following the manufacturer's instructions.

### 2.6. Antibacterial Testing: INT Colorimetric Assay

#### 2.6.1. MIC Determination

The MICs of the aqueous and organic extracts from the barks of cinnamon, buds of clove, and leaves of thyme were determined using a rapid INT colorimetric assay [8, 12]. First, the extracts were weighed and dissolved in DMSO. Stock solutions of the extracts and a reference antibiotic were prepared at concentrations of 4096 μg/mL and 2048 μg/mL, respectively. The resulting solutions were then added to MHB and serially diluted twofold in a 96-well microplate. Therefore, the concentrations of test extracts ranged from 1024 to 8 μg/mL and from 512 to 4 μg/mL for the reference antibiotic (TET). The inoculum was prepared from bacterial colonies that were 18–24 h old. Bacteria were collected and introduced into 10 mL of distilled water. This solution was compared to a standard 0.5 McFarland solution to ensure a bacterial suspension with a concentration of 1.5 × 10^8^ CFU/mL. This suspension was then diluted in MHB to achieve an inoculum concentration of 2 × 10^6^ CFU/mL. One hundred microliters of the prepared inoculum in MHB were added to each microplate. After adding the inoculum, the plates were covered with a sterile plate sealer and agitated on a shaker to mix the contents of the wells. The plates were then incubated at 35°C–37°C for 18–24 h. DMSO control wells were tested for baseline color interference with INT. Its final concentration was kept below 2.5%, which did not affect microbial growth, nor interact with INT. Wells containing only MHB, 100 μL of inoculum, and DMSO at a final concentration of 2.5% served as the negative control. TET was used as a reference antibiotic (positive control), and MHB alone served as the neutral control. The MICs of each extract were determined after the 18–24-h incubation at 35°C–37°C, following the addition of 40 μL of INT (0.2 mg/mL) and a further incubation at 35°C–37°C for 30 min. Viable bacteria reduced the yellow tetrazolium (INT) dye to pink. The MIC for each sample was defined as the lowest concentration that prevented any color change upon visual observation, indicating no bacterial growth or activity.

#### 2.6.2. MBC Determination

The MBC was determined by pipetting 50 μL of samples from the wells containing concentrations above the MIC into new plates that contained 150 μL of MHB in each well. The plates were incubated at 35°C–37°C for 48 h, after which 40 μL of INT was added, as previously described. The MBC was defined as the lowest concentration of the extract that resulted in no bacterial growth, indicated by the absence of color change (yellow tetrazolium (INT) dye to pink). Each assay was conducted independently three times, with duplicate measurements. In cases where the results varied, the MIC or MBC was reported as the value that occurred most frequently.

### 2.7. Interactive Antimicrobial Profile of Plant Extracts Combined With Antibiotics: The Checkerboard Assay

**Figure 1:**
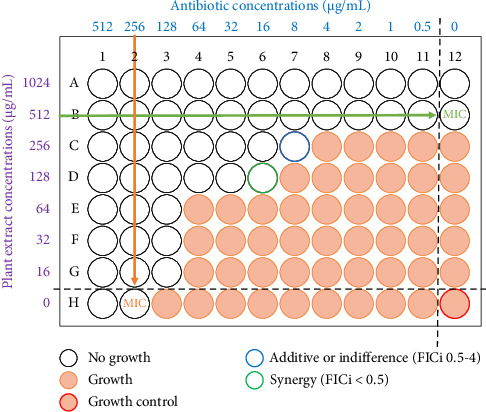
Checkerboard assay layout for combination testing between plant extract and antibiotics. Columns 1–11 contain 2-fold serial dilutions of antibiotics (ATB), and rows A–G contain 2-fold serial dilutions of plant extract (PE). Column 12 contains a serial dilution of PE alone, while row H contains a serial dilution of ATB alone. These controls are used to determine the MIC value for each test compound, which in turn are used to calculate the FIC and FICi values using formulas (2)–(4). The concentrations of ATB and PE in well D6 resulted in a synergistic effect (FICi < 0.5), while in well C7, the effect was additive or indifferent (FICi = 0.5–4).

The combination of extracts at sub-inhibitory concentrations with antibiotics (TET and ERY at 512 μg/mL and CIP at 128 μg/mL) was evaluated using the checkerboard synergy assay, following the method previously described for determining MIC. The key difference was that successive dilutions were made in the presence of antibiotics, with 50 μL of extracts at sub-inhibitory concentrations added to each well. The total volume in each well was brought to 200 μL by adding 100 μL of bacterial inoculum at a density of 4 × 10^6^ CFU/mL. Stock solutions of the antibiotics and plant extracts were prepared by dissolving them in DMSO and then further diluting them in MHB. Working solutions of each test substance were made by diluting the stock solutions in MHB within separate microtiter plates. The antibiotics were serially diluted across 11 columns (1–11), with concentrations decreasing from wells 1 to 11 of each row, at a volume of 100 μL per well. Similarly, the extracts were diluted in seven rows (A–G), with concentrations reducing from wells A to G of each column. The checkerboard assay was then set up on a fresh plate by transferring 50 μL of the diluted antibiotic into the corresponding well of the extract, followed by the addition of 100 μL of the bacterial suspension. Each antibiotic and extract alone were included in row H and column 12, respectively, to determine their MICs. A column of wells containing only the antibiotic and inoculum, without the extract, served as the positive control. The checkerboard setup is illustrated in Figure 1. The plates were incubated at 35°C–37°C for 18–24 h, and the MICs were measured using the INT method. The interaction between the combined extracts and the standard drugs was analyzed by calculating the fractional inhibition concentration index (FICi) based on MIC measurements, using the specified formulas (2)–(4) [36, 37]

(2) $$\begin{array}{c} \text{FICextract}=\frac{\text{MIC of extract in combination}}{\text{MIC of extract alone}}, \end{array}$$

(3) $$\begin{array}{c} \text{FICantibiotic}=\frac{\text{MIC of antibiotic in combination}}{\text{MIC of antibiotic alone}}, \end{array}$$

(4) $$\begin{array}{c} \text{FICi}=\text{FICextract}+\text{FICantibiotic}. \end{array}$$

### 2.8. Data Analysis and Interpretation

All assays were performed in duplicate (two replicates per experimental condition) and repeated thrice to ensure their reproducibility. The data were analyzed using a one-way ANOVA to compare the means of different groups. Following this, Tukey's test was employed for multiple comparisons to identify specific significant differences between the groups. A significance level of *p* < 0.05 was established for these analyses. Data preparation was conducted in Excel 2016 before being transferred to GraphPad Prism 8.0.2 for analysis. The cutoff points for the classification of antibacterial agents from natural sources were considered as follows: outstanding activity when MIC ≤ 8 μg/mL; excellent activity when 8 < MIC ≤ 64 μg/mL; very good activity when 64 < MIC ≤ 128 μg/mL; good activity when 128 < MIC ≤ 256 μg/mL, average activity when 256 < MIC ≤ 512 μg/mL, weak activity when 512 < MIC ≤ 1024 μg/mL [38]. The MBC/MIC < 4 indicates a bactericidal effect, whereas MBC/MIC > 4 suggests a bacteriostatic effect [39]. The resulting interactions were interpreted as synergistic when FICi ≤ 0.5; additive when 0.5 < FICi ≤ 1, indifference when 1 < FICi ≤ 4, and antagonism when FICi > 4 [40].

## 3. Results

### 3.1. Extraction Yield

The results of the percentage yield for the aqueous and organic extracts of the studied plants are presented in Table 2. It is important to note that the extraction yield varied depending on the type of solvent used. Overall, the best extraction yield was observed with 70% ethanol; however, this solvent was less effective for thyme, yielding only 3%, compared to the water extract, which achieved a yield of 9.61%. The highest extraction yield was obtained from the hydroethanolic extract of cinnamon, which reached 29.08%. Additionally, both solvents (ethanol 70% and water) produced similar yields of 20% for clove.

### 3.2. Phytochemical Analysis

The qualitative phytochemical screening assessed seven major classes of secondary metabolites found in plants: phenols, flavonoids, saponins, terpenoids, anthocyanins, tannins, and alkaloids. Phenols, terpenoids, and anthocyanins were detected in all extracts, while the aqueous extracts from clove and thyme contained all classes of screened phytochemicals. In contrast, the aqueous extract of cinnamon contained the fewest classes of secondary metabolites, as saponins, tannins, and alkaloids were not identified. All herbal extracts contained flavonoids, except for the organic extract from thyme. Likewise, neither extract of cinnamon showed the presence of saponins (Table 3).

### 3.3. Antibacterial Activity of Test Herbals Alone

The antibacterial activities of aqueous and hydroethanolic extracts of cinnamon, clove, and thyme were evaluated using MIC and MBC against 14 strains and clinical isolates of both Gram-negative and Gram-positive bacteria. Overall, the antibacterial effectiveness of the herbal extracts varied depending on the type of bacteria and the solvent used, with MIC values ranging from 13.33 ± 2.67 to 1024 ± 0.00 μg/mL. The extracts displayed inhibitory potential against all tested pathogens, but the degree of potency varied. The aqueous and organic extracts of clove were found to be more potent, with most MIC values falling between 13.33 ± 2.67 and 256 ± 0.00 μg/mL. The efficacy of clove extracts was significantly higher (*p* < 0.05) compared to the other herbal extracts studied on the majority of the studied microorganisms. Notably, both extracts demonstrated very good activity [38], with MIC < 100 μg/mL recorded for five pathogens: *S. aureus* SANR 46003, *S. Typhimurium* STM CPC, *S. typhi* ST CPC, *S. dysenteriae* SD CPC, and *S. flexneri* SFNR 518 for the aqueous extract. The hydroethanolic extract exhibited similar efficacy against three pathogens: *S. aureus* SANR 46003, *S. typhimurium* STM CPC, and *S. flexneri* SFNR 518. Most of these activities (MIC < 100 μg/mL) from both clove extracts were observed on the same strains or clinical isolates, including *S. aureus* SANR46003, *S. typhimurium* STM CPC, and *S. flexneri* SFNR 518. The MIC reported for clove extracts was lower (indicating better activity) compared to the reference antibiotic (TET) in some cases. Specifically, this was observed for *S. aureus* SANR 46003 with both the aqueous and ethanol clove extracts, as well as for the ethanol extract of cinnamon. Additionally, *S. flexneri* SFNR 518 showed similar results with both the aqueous and ethanol clove extracts and the aqueous extract of cinnamon. However, no significant differences were found (*p* < 0.05). The aqueous extract of clove also exhibited comparable efficacy to the reference antibiotic for *S. enteritidis* SENR 13555 and *S. typhimurium* ATCC 14028, as well as for *S. typhi* ST CPC (Table 4). The aqueous and organic extracts of clove also displayed an MBC/MIC ≤ 4 against 8 and 10 out of the 14 studied bacterial strains, respectively, comparable to that of the reference antibiotic in general (Table 5). This effect was less pronounced with the other herbal extracts, which exhibited either no effect or a high MBC (Table 6) relative to their respective MICs. However, the cinnamon hydroethanolic extract showed MIC equal to MBC (MBC/MIC = 1.33 ± 0.33) against *S. dysenteriae* SD CPC, where data were significantly different (*p* < 0.05) compared to other extracts and the reference antibiotic on the same strain. Similar results (*p* < 0.05) were observed with both thyme extracts against *S. enteritidis* SENR 13555 (Table 5).

It is noteworthy that all clinical isolates from RHB exhibited the highest resistance profile toward both the herbal extracts and the reference drug. Consequently, these isolates have been selected for further interactive antimicrobial assessment involving a combination of the most active plant (clove) and selected traditional antibiotics.

### 3.4. Interactive Antimicrobial Activity of Clove Extracts With Standard Antibiotics

**Figure 2:**
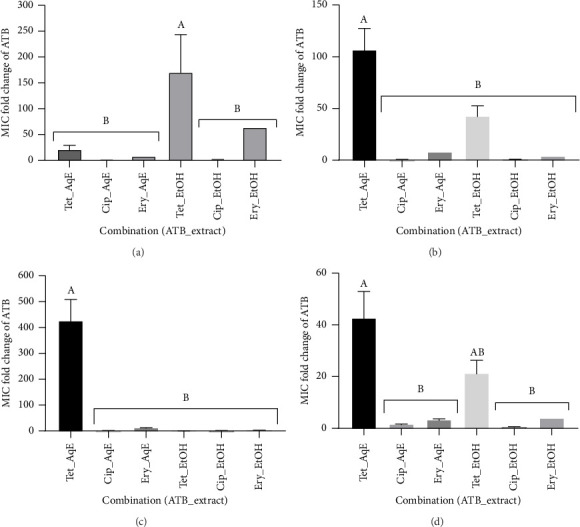
MIC fold change of antibiotics in combination with aqueous (AqE) and ethanol 70% (EtOH) extracts of clove combined with tetracycline, ciprofloxacin, and erythromycin on *E. coli* EC-RHB (a), *S. typhi* ST-RHB (b), *S. aureus* SA-RHB (c), and *P. aeruginosa* PA-RHB (d). The most important reduction in the MIC of antibiotics was recorded with both clove aqueous (up to 500-fold reduction in the MIC of antibiotic) and ethanol (up to 170-fold reduction in MIC of the antibiotic) extracts combined with tetracycline, suggesting marked potentiation effects of test clove extracts. Values are expressed as mean ± SEM (standard error of the mean). Groups with different letters (A-B) are considered statistically different (*p* < 0.05) using Tukey's multiple comparison test. TET: tetracycline, CIP: ciprofloxacin, ERY: erythromycin, and ATB: antibiotic.

The types and effects of interactions between the most active extracts—aqueous (AqE) and ethanolic (EtOH) from clove—on the MICs of selected antibiotics are presented in Table 7. When tested alone, the MICs of the antibiotics varied from 6.67 ± 1.333 to 426.7 ± 85.33 μg/mL, confirming the MDR nature of the studied bacteria. The MICs of TET, ERY, and CIP were significantly reduced (*p* < 0.05) in combination with the extracts compared to when they were tested alone (Table 7). The interactions observed were more synergistic than additive, with 12 synergistic effects identified out of the 24 combinations tested, determined by a FICi < 0.5 [40]. Significant reductions in MICs of antibiotics were noted with the following combinations, as illustrated in Figure 2: AqE-TET (21.33 ± 5.33-fold) and EtOH-TET (170.7 ± 42.67-fold) against *E. coli* EC-RHB; AqE-TET (106.7 ± 21.33-fold) and EtOH-TET (42.67 ± 10.67-fold) against *S. typhi* ST-RHB; AqE-TET (426.7 ± 85.33-fold) and AqE-ERY (13.33 ± 2.67-fold) against *S. aureus* SA-RHB; and AqE-TET (42.67 ± 10.67-fold) and EtOH-TET (21.33 ± 5.33-fold) against *P. aeruginosa* PA-RHB. It was observed that the AqE-TET exhibited a significantly greater reduction (*p* < 0.05) compared to other combinations across all bacterial strains, except for *E. coli* (EC-RHB), where the EtOH-TET showed the most significant fold change (*p* < 0.05).

**Figure 3:**
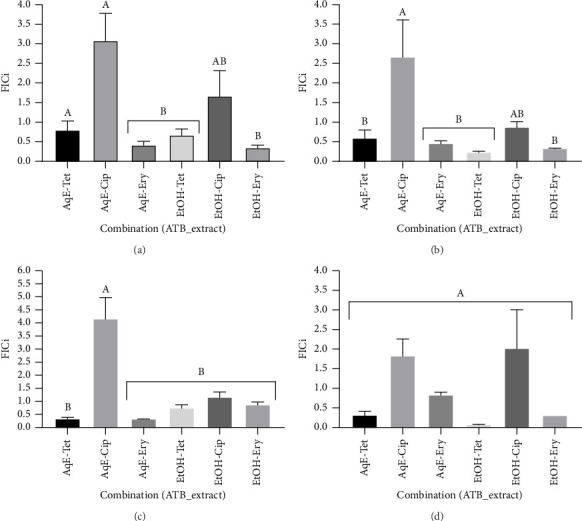
FICi values of aqueous (AqE) and ethanol 70% (EtOH) extracts of clove combined with tetracycline, ciprofloxacin, and erythromycin on *E. coli* EC-RHB (a), *S. typhi* ST-RHB (b), *S. aureus* SA-RHB (c), and *P. aeruginosa* PA-RHB (d). The most notable synergistic interaction was observed with EtOH-TET (FICi = 0.078 ± 0.016) against *P. aeruginosa* (PA-RHB). The most promising interactions suggesting either synergy or additive effects were observed in combinations that included TET and ERY. Values are expressed as mean ± SEM (standard error of the mean). Groups with different letters (A-B) are considered statistically different (*p* < 0.05) using Tukey's multiple comparison test. TET: tetracycline, CIP: ciprofloxacin, ERY: erythromycin, ATB: antibiotic, and FICi: fractional inhibitory concentration index. Synergy was considered when FICi < 0.5.

Figure 3 presents the FICi recorded for various combinations. The most notable synergistic interaction was observed with the EtOH-TET (FICi = 0.078 ± 0.016) against *P. aeruginosa* (PA-RHB). However, the results were not statistically different from the FICi values of other combinations against the same strain. Most FICi values for combinations involving TET and ERY were significantly lower (*p* < 0.05) than those for the combination with CIP (Figure 3). This suggests that the most promising results of the study, suggesting either synergy or additive effects, were observed in combinations that included TET and ERY. Conversely, the study found poor interactions with CIP. The only exception was the combination of EtOH-CIP, which demonstrated an additive effect against *E. coli* EC-RHB. All other combinations involving CIP displayed either indifference or antagonism. Indeed, the only instance of antagonism was observed with AqE-CIP against *S. aureus* SA-RHB, which had a FICi of 4.17 ± 0.83. This value is significantly different (*p* < 0.05) from the FICi of all other combinations, as shown in Figure 3(c).

## 4. Discussion

Despite significant advancements in scientific knowledge and medicine, infectious diseases continue to be a leading cause of illness and death worldwide. They account for approximately half of all deaths in tropical countries [41]. The increase of drug-resistant pathogens, especially bacteria, is complicating their treatment and control. The infectious diseases caused by bacteria included in this study are among the major contributors to morbidity and mortality in sub-Saharan Africa. These pathogens have been classified by the WHO within the high-priority pathogen group [2], highlighting the relevance of this research. Various strategies are being explored to address these challenging bacterial infections [42, 43]. One significant alternative is utilizing medicinal plants, either individually or in combination [7]. Herbal teas or beverages are increasingly gaining attention as natural products in the context of increasing antibiotic resistance [18]. In many societies, including Cameroon, tea-drinking rituals and customs play a significant role in social gatherings and cultural events. Furthermore, these practices are deeply embedded in traditional medicine systems, reflecting their long history of use for various ailments and promoting health [18, 21]. From a public health perspective, herbal teas offer potential benefits like antioxidant and anti-inflammatory effects, contributing to overall well-being and disease prevention [18]. This study examined the antibacterial potential of herbal beverages and organic extracts derived from cinnamon, clove, and thyme, as well as the combined antimicrobial effects of the most active extracts (clove) with selected traditional antibiotics. The assays were conducted against 14 strains and clinical isolates of multidrug-resistant bacteria. The antibacterial assay (MIC) revealed notable inhibitory effects from the tested herbs. All extracts demonstrated inhibitory effects (MIC) against all tested bacterial strains, with potency varying depending on the specific species or strains of bacteria (Table 4). The MICs recorded ranged from 13.33 ± 2.67 to 1024 ± 0.00 μg/mL, which can be categorized as exhibiting excellent to weak activity based on established thresholds for evaluating the antibacterial efficacy of botanicals [38]. These findings are particularly relevant given the drug-resistant nature of the studied microorganisms. Variations in antibacterial activities among the different herbal beverage extracts and organic extracts can be attributed to the specific bacterial strain or type of extract. This is supported by the study conducted by Hacioglu et al. [18], which examined the in vitro antimicrobial activities of different herbal teas against challenging bacteria. Indeed, the sensitivity of bacterial species to therapeutic agents can vary due to several factors, including natural physiology, cell wall structure, and the presence of specific proteins. Furthermore, bacteria can express multiple strategies to evade the effects of drugs to varying degrees, such as enzymatic inactivation, altered drug targets, reduced permeability, or the use of efflux pumps [43]. Additionally, the differences in antibacterial potency among the tested herbal extracts can be attributed to the types and concentrations of bioactive metabolites present [7]. The observed antibacterial effects also correspond with the traditional uses of these selected herbs in managing infectious diseases caused by the studied pathogens, specifically infections related to *Salmonella*, *Shigella*, and *Staphylococcus*.

The aqueous and hydroethanolic extracts from clove demonstrated notable antibacterial potency, with most MIC values ranging between 13.33 ± 2.67 and 256 ± 0.00 μg/mL, indicating excellent to good activity [38]. Furthermore, MICs < 100 μg/mL were observed for five pathogens with clove aqueous extract and three pathogens with the hydroethanol extract (Table 4). Notably, those significant activities for both extracts were recorded against the same strains or clinical isolates, including *S. aureus* SANR46003, *S. typhimurium* STM CPC, and *S. flexneri* SFNR 518. These bacteria demonstrate a notable resistance profile and are frequently associated with common difficult-to-treat infections, such as skin and gastrointestinal disorders [2]. Therefore, our findings hold significant potential for advancing clinical strategies in the fight against these pathogens. Furthermore, the activity of both clove extracts was comparable to that of the reference antibiotic, TET. The extraction yield for both solvents was similar (Table 2). This leads us to hypothesize that their comparable antibacterial properties may stem from the presence of similar compounds in similar amounts. Additionally, this finding suggests that both water and 70% ethanol serve as effective extraction solvents for clove. These results underscore the potent antibacterial properties of clove and highlight their potential applications, especially against infections caused by *S. aureus*, *S. typhimurium*, and *S. flexneri*. Both aqueous and hydroethanolic extracts of clove showed MBC/MIC ≤ 4 against most bacterial strains studied, with 50% effectiveness for aqueous extracts and 64% for ethanol extracts, suggesting bactericidal effects. This indicates that the extracts can achieve drug concentrations capable of eliminating 99.9% of the targeted organisms at the recorded MICs. Conversely, a high MBC/MIC ratio (MBC/MIC > 4) implies that it may be challenging to safely deliver enough of the antibacterial to eliminate 99.9% of the bacteria, classifying the agent as bacteriostatic [39].

While previous studies have reported on the antibacterial properties of the studied plants, few have examined the extracts from herbal beverages, as discussed in this work. Most past research has focused on their essential oils. For instance, the essential oils of clove, thyme, and cinnamon have shown potent antibacterial activity against a variety of MDR bacterial strains, including *S. aureus*, *E. coli*, and *P. aeruginosa*, which effectiveness was attributed to the presence of bioactive compounds such as eugenol, thymol, and cinnamaldehyde, respectively [31, 32]. Although past research has largely focused on their essential oils, there is a significant opportunity to explore beverage-prepared extracts made with water and/or ethanol solvents. These extracts bring several advantages, including safety, accessibility, and general acceptability. Additionally, herbal teas serve as a widely used dosage form in traditional medicine, highlighting their importance and potential in natural health practices. Research on herbal teas and their components remains limited, particularly concerning reports on herbal organic extracts as examined in the current study. However, some previous studies have demonstrated their remarkable ability to combat various diseases, including drug-resistant bacterial infections [25]. For instance, thyme (*T. vulgaris*) extracts demonstrated strong antibacterial effects against methicillin-resistant *S. aureus* (MRSA) and MDR *Acinetobacter baumannii,* with MIC values between 78 and 312 μg/mL [25]. In the current study, aqueous and hydroethanol extracts had MIC values of 256–512 μg/mL against *S. aureus* SANR. Hacioglu et al. [18] found that 31 herbal tea infusions were effective against various microorganisms, while Venkatesan et al. [23] highlighted the antibacterial properties of *H. sabdariffa* L. (rosella) tea. Additionally, Alkufeidy et al. [25] reported that green tea extracts showed activity against acne-causing bacteria. Further reviews have underscored the nutraceutical potential of herbal beverages in managing various health issues, including infectious diseases [26]. The findings regarding aqueous herbal teas and other solvent extracts indicate a promising pathway for identifying effective agents against both sensitive and drug-resistant bacteria. Notably, these herbal preparations are gaining significant acceptance among the population.

Different plant extracts exhibit varying antibacterial activities, which may be attributed to the presence of diverse phytoconstituents, including phenolics, terpenoids, tannins, anthocyanins, saponins, and alkaloids, as shown in Table 3. Phenols, terpenoids, and anthocyanins were consistently detected in all studied extracts, highlighting their prevalence across various plant sources. The aqueous extracts from clove and thyme were particularly notable, as they contained a complete range of the screened phytochemical classes. In contrast, the aqueous extract of cinnamon provided an interesting insight, as it included the fewest classes of secondary metabolites, lacking saponins, tannins, and alkaloids. This variation underscores the importance of the extraction method and the specific plant part analyzed, as different solvents are capable of dissolving different types of secondary metabolites. Additionally, certain metabolites may be more concentrated in specific tissues or organs of the plant. Understanding these factors can help optimize the extraction process and enhance the identification of beneficial compounds in future studies. Each of these phytoconstituents possesses a unique and complex mode of action contributing to their antibacterial properties [44]. While a single, definitive mechanism for the antibacterial activity of plant extracts or their derived constituents has yet to be established, it is believed that various groups of compounds may target cell structure, ions, other active cell sites, and critical molecules found inside the cell such as proteins and DNA, leading to antibacterial effects [7]. Clove extract, in particular, has been reported to have a high concentration of phenolic compounds [45]. These active phenolic compounds may play a significant role in the enhanced antibacterial activity of the extract by altering membrane permeability, blocking efflux pumps, and inducing protein denaturation in microorganisms [46]. However, further screening and identification of phytoconstituents, followed by deep mechanistic investigations, are required for confirmation.

Antimicrobial agents are among the most commonly prescribed medications, and there is a considerable likelihood of concurrent use with popular herbal beverages [20], such as clove tea, which is widely consumed in Cameroon as a spice or decoction to prevent or manage various ailments, including bacterial infections. Herbal teas and beverages are increasingly being recognized as components of alternative or complementary medicine, serving as either standalone treatments or as adjuvants in antimicrobial therapy [7, 18]. Combining antibiotics with herbal teas may be particularly beneficial for treating severe infections, as it can enhance the effectiveness of the antibiotics and help prevent or delay the development of resistance (this is known as pharmacodynamics synergy) [18]. Additionally, herbal teas may influence the pharmacokinetics of the antibiotics, which involves how the drugs are absorbed, distributed, metabolized, and excreted by the body. For example, clove extract can inhibit a liver enzyme that is responsible for metabolizing antibiotics. This inhibition may reduce the breakdown of the antibiotic, potentially resulting in increased blood levels and a longer duration of effectiveness. The ideal targets for combination therapy include achieving synergistic drug interactions, preventing resistance, and minimizing toxicity and costs. Conversely, antagonistic interactions can be disadvantageous, as they may render the combination less effective than a single drug. The present study revealed noteworthy synergistic interactions between the aqueous or hydroethanolic extract of clove and specific antibiotics (particularly TET and ERY) against all selected MDR clinical isolates of *E. coli*, *S. aureus*, *S. typhi*, and *P. aeruginosa*, as illustrated in Table 7. The most promising synergistic interaction (FICi = 0.078 ± 0.016) was recorded with the EtOH-TET against *P. aeruginosa* (PA-RHB) (Figure 3). In some scenarios, we observed reductions of more than 100-fold in the MICs of antibiotics (Table 7). The MIC fold change of TET with the clove ethanol extract was 171 ± 42.67 against *E. coli* EC RHB, 107 ± 21.33 in combination with clove aqueous extract against *S. typhi* ST RHB, and 427 ± 85.33 against *S. aureus* SA RHB with clove aqueous extract, which values were statistically different (*p* < 0.05) in comparison with other combinations (Figure 2). These combinations, particularly with TET, may serve as effective and safe alternatives to antibiotic monotherapy or as adjuncts in combination therapy with plant extracts. Our findings indicate that incorporating plant extracts can allow for a reduction in antibiotic dosages, potentially decreasing the risk of developing resistance in bacteria. However, we reported a case of antagonism when combining the aqueous extract of clove with CIP on *S. aureus* SA-RHB, highlighting the need for caution in these combinations. A previous investigation reported similar results where the antagonistic interaction with herbal teas, including rosehip and pomegranate blossom teas, was reported in combination with CIP on MDR bacteria [18]. A possible explanation for the observed antagonism may lie in the differences between the antibacterial effects of the agents involved, specifically, the distinction between bacteriostatic and bactericidal effects. A bacteriostatic agent can interfere with the effectiveness of a bactericidal agent [47]. For instance, the aqueous extract of clove demonstrated a bacteriostatic effect (Table 5), while CIP produced a bactericidal effect against *S. aureus* SA-RHB. This means that the clove aqueous extract may have hindered CIP's ability to kill the bacteria, resulting in an antagonistic effect. Furthermore, the aqueous extract may slow down or halt bacterial growth, which is essential for bactericidal drugs like CIP to effectively eliminate bacteria. It could also alter bacterial metabolism, impacting the effectiveness of other antibiotics [47]. Therefore, in situations where antagonism is a concern, clinicians might consider alternative strategies, such as using single-drug therapy or exploring different combinations that demonstrate synergistic effects. Clove may disrupt bacterial membranes or inhibit efflux pumps due to its phenolic content, while TET and ERY work by inhibiting bacterial protein synthesis. The simultaneous activity of clove and these antibiotics at their respective active sites may lead to a significant reduction in the MICs of the antibiotics. Further molecular investigations are essential for deeper understanding. Previous studies have noted the antibiotic-resistance-modifying properties of clove but primarily focused on essential oils [48, 49]. Previous studies have also examined the effectiveness of herbal teas combined with antibiotics against resistant bacteria. Hacioglu et al. [18] found that interactions (synergistic, additive, antagonistic) varied between herbal teas and commonly used antibiotics, with synergy most commonly observed with ampicillin or nystatin. In contrast, combinations with CIP or ERY were often antagonistic [18]. Another study on *M. oleifera* tea extracts showed significant reductions in antibiotic MICs against *K. pneumoniae*, *E. coli*, and *S. aureus* [24]. Alkufeidy et al. [25] found that green tea extract and catechin improved the effectiveness of antibiotics against acne-causing bacteria. Overall, reports regarding aqueous herbal teas and solvent extracts suggest a promising avenue for discovering effective agents against both sensitive and drug-resistant bacteria. It suggests that incorporating herbal beverages with standard antibiotics could address the MDR issue. This lays the groundwork for future in vivo studies to determine the dose–effect relationship of these combinations in treating infections caused by MDR bacteria such as *E. coli*, *S. aureus*, *S. typhi*, and *P. aeruginosa*.

## 5. Conclusion

The current study provides in vitro scientific evidence demonstrating the effectiveness of clove, cinnamon, and thyme against drug-resistant pathogens such as *S. aureus*, *E. coli*, *Salmonella* species, *Shigella* species, and *P. aeruginosa*. The results indicate a significant efficacy of aqueous and hydroethanolic extracts of clove. In combination assay, clove extracts (water and 70% ethanol) exhibited a marked synergy with TET and ERY. However, an antagonistic effect was observed with clove aqueous extract and CIP on *S. aureus*. The findings underscore the importance of further research into herbal beverages, both alone and in combination with conventional antibiotics, for treating microbial infections. This approach may lead to minimal or no side effects while reducing the emergence of antibiotic resistance. To better understand the antagonistic effects and determine the specific contexts in which they may reduce therapeutic efficacy, more detailed investigations are needed. Furthermore, our study currently lacks investigations in animal models, an assessment of the molecular mechanisms underlying antibacterial activity, and an exploration of the variability observed among bacterial strains. These areas will be addressed in future research.

## Figures and Tables

**Table 1:** Phytochemical screening tests.

Secondary metabolites	Test type and reagents	Positive results (presence)
Tannins	5 mg of extract dissolved in methanol, then an addition of 5 drops of 0.5% H_2_SO_4_	Green to bluish-black color
Saponins	5 mg of extract + 5 mL of distilled water, heating for 5 min, cooling, then shake vertically for 15 s and leave to rest	Formation of persistent foam
Phenols	2 mL of diluted extract in dimethylsulfoxide (DMSO) was placed in a clean test tube and 3-4 drops of 10% FeCl_3_ were added	Formation of a dark green-to-black color
Flavonoid	2 mL of diluted extract in DMSO was introduced in a clean test tube and a few drops of lead acetate were added	Appearance of a golden yellow precipitate
Alkaloids	1 mL of diluted extract in DMSO was introduced in a clean test tube + 1 mL of 1% H_2_SO_4_ boiled for 5 min, then allowed to cool + 5 drops of Mayer's reagent	Appearance of a white precipitate
Anthocyanin	2 mL of diluted extract in DMSO was introduced in a clean test tube + 1% H_2_SO_4_ and was heated for 5 min	Formation of a deep yellow-to-orange color
Terpenoids	2 mL of diluted extract in DMSO was introduced in a clean test tube + 3–5 drops of 5% concentrated H_2_SO_4_ were added + 3 drops of methyl chloride (CH_2_Cl_2_) were added and stirred	Formation of green to golden yellow

**Table 2:** Percentage yield of extracts.

Plants	Extracts	Weight of dry powder (g)	Weight of extract (g)	Color	Texture	Percentage yield (%)
Clove	AqE-decoction	50	9.77	Dark brown	Waxy	19.54
	EtOH	50	10.32	Brick red	Waxy	20.64

Cinnamon	AqE-decoction	50	5.08	Reddish-brown	Waxy	10.16
	EtOH	50	14.54	Brick red	Waxy	29.08

Thyme	AqE-infusion	100	9.61	Greenish	Flaky	9.61
	EtOH	100	3	Greenish	Flaky	3

*Note:* EtOH = ethanol 70%.

Abbreviation: AqE = aqueous extract.

**Table 3:** Major classes of secondary metabolites identified in the studied extracts.

Plants	Extracts	Classes of screened phytochemicals						
		Phenolic compounds	Flavonoids	Saponins	Terpenoids	Anthocyanins	Tannins	Alkaloids
Cinnamon	AqE-decoction	+	+	−	+	+	−	−
	EtOH	+	+	−	+	+	+	+

Clove	AqE-decoction	+	+	+	+	+	+	+
	EtOH	+	+	−	+	+	+	+

Thyme	AqE-infusion	+	+	+	+	+	+	+
	EtOH	+	−	+	+	+	+	+

*Note:* +: presence. −: absence. EtOH = ethanol 70%.

Abbreviation: AqE = aqueous extract.

**Table 4:** Minimum inhibitory concentrations (MIC in μg/mL) of the studied herbals.

Bacteria strains/isolates		MIC (in μg/mL)						
		Clove		Cinnamon		Thyme		Antibiotic
		AqE	EtOH	AqE	EtOH	AqE	EtOH	TET
*S. aureus*	SANR 46003	42.67 ± 10.67^c^	85.33 ± 21.33^c^	1024 ± 0.00^a^	26.67 ± 5.33^c^	341.3 ± 85.33^b^	256 ± 0.00^b^	128 ± 0.00^bc^
	SA RHB	128 ± 0.00^b^	106.7 ± 21.33^b^	1024 ± 0.00^a^	1024 ± 0.00^a^	1024 ± 0.00^a^	1024 ± 0.00^a^	53.33 ± 10.67^c^

*S. enteridis*	SENR 13555	213.3 ± 42.67^b^	341.3 ± 85.33^b^	1024 ± 0.00^a^	341.3 ± 85.33^b^	1024 ± 0.00^a^	341.3 ± 85.33^b^	128 ± 0.00^b^
	SE CPC	341.3 ± 85.33^b^	512.0 ± 256^ab^	106.7 ± 21.33^b^	1024 ± 0.00^a^	1024 ± 0.00^a^	1024 ± 0.00^a^	3.33 ± 0.67^c^

*S. typhimurium*	ATCC 14028	213.3 ± 42.67^b^	213.3 ± 42.67^b^	426.7 ± 85.33^b^	1024 ± 0.00^a^	853.3 ± 171^a^	1024 ± 0.00^a^	32 ± 0.00^bc^
	STM CPC	53.33 ± 10.67^c^	64 ± 0.00^c^	213.3 ± 42.67^b^	256 ± 0.00^b^	1024 ± 0.00^a^	1024 ± 0.00^a^	4 ± 0.00^d^

*S. paratyphi* B	SPT-B CPC	171 ± 42.67^b^	341.3 ± 85.33^b^	1024 ± 0.00^a^	1024 ± 0.00^a^	1024 ± 0.00^a^	171 ± 42.67^b^	3.33 ± 0.67^c^

*S. typhi*	ST CPC	64 ± 0.00^c^	256 ± 0.00^b^	1024 ± 0.00^a^	1024 ± 0.00^a^	1024 ± 0.00^a^	1024 ± 0.00^a^	53.33 ± 10.67^c^
	ST RHB	853.3 ± 171^a^	1024 ± 0.00^a^	1024 ± 0.00^a^	1024 ± 0.00^a^	1024 ± 0.00^a^	1024 ± 0.00^a^	426.7 ± 85.33^b^

*S. dysenteriae*	SD CPC	53.33 ± 10.67^b^	106.7 ± 21.33^b^	341.3 ± 85.33^a^	106.7 ± 21.33^b^	170.7 ± 42.67^a^	128 ± 0.00^b^	4 ± 0.00^c^

*S. flexneri*	SFNR 518	21.33 ± 5.33^c^	13.33 ± 2.67^c^	85.33 ± 21.33^c^	256 ± 0.00^b^	1024 ± 0.00^a^	341.3 ± 85.33^b^	128 ± 0.00^bc^

*E. coli*	ATCC 25922	128 ± 0.00^c^	512 ± 0.00^b^	213.3 ± 42.67^c^	1024 ± 0.00^a^	1024 ± 0.00^a^	853.3 ± 171^ab^	171 ± 42.67^bc^
	EC RHB	341.3 ± 85.33^b^	106.7 ± 21.33^c^	1024 ± 0.00^a^	1024 ± 0.00^a^	1024 ± 0.00^a^	1024 ± 0.00^a^	256 ± 0.00^bc^

*P. aeruginosa*	PA RHB	853.3 ± 171^a^	1024 ± 0.00^a^	1024 ± 0.00^a^	1024 ± 0.00^a^	1024 ± 0.00^a^	1024 ± 0.00^a^	341.3 ± 85.33^b^

*Note:* Plant extracts were tested at 1024 μg/mL and the reference antibiotic (TET: tetracycline) at 512 μg/mL. EtOH: ethanol 70% extract. MIC values in bold: excellent activities (MIC < 100 μg/mL). Values are expressed as mean ± SEM (standard error of the mean). Groups with different letters (a–c) are considered statistically different (*p* < 0.05) using Tukey's multiple comparisons test.

Abbreviations: AqE = aqueous extract; MIC = minimum inhibitory concentration.

**Table 5:** MBC/MIC ratio of the studied herbals.

Bacteria strains/isolates		MBC/MIC^∗^ ratio						
		Clove		Cinnamon		Thyme		Antibiotic
		AqE	EtOH	AqE	EtOH	AqE	EtOH	TET
*S. aureus*	SANR 46003	16 ± 0.00^a^	4 ± 0.00^b^	—	13.33 ± 2.67^a^	3.33 ± 0.67^b^	3.33 ± 0.67^b^	1.67 ± 0.33^b^
	SA RHB	4 ± 0.00^a^	4 ± 0.00^a^	—	—	—	—	8 ± 0.00^a^

*S. enteridis*	SENR 13555	3.33 ± 0.67^a^	3.33 ± 0.67^a^	—	3.33 ± 0.67^a^	1 ± 0.00^b^	1.33 ± 0.33^b^	4 ± 0.00^a^
	SE CPC	2 ± 0.00^b^	1.67 ± 0.33^b^	8 ± 0.00^a^	—	—	—	8 ± 0.00^a^

*S. typhimurium*	ATCC 14028	2.67 ± 0.67^ab^	4 ± 0.00^a^	2 ± 0.00^b^	—	—	—	1.67 ± 0.33^b^
	STM CPC	4 ± 0.00^ab^	6.67 ± 1.33^a^	4 ± 0.00^ab^	3.33 ± 0.67^b^	—	—	1.67 ± 0.33^b^

*S. paratyphi* B	SPT-B CPC	4.67 ± 1.764^ab^	1.67 ± 0.33^b^	—	—	—	3.33 ± 0.67^b^	8 ± 0.00^a^

*S. typhi*	ST CPC	10.67 ± 2.67^a^	3.33 ± 0.67^b^	—	—	—	—	4 ± 0.00^ab^
	ST RHB	—	—	—	—	—	—	—

*S. dysenteriae*	SD CPC	6.67 ± 1.33^a^	4 ± 0.00^a^	3.00 ± 1.00^ab^	1.33 ± 0.33^b^	6.67 ± 1.33^a^	4 ± 0.00^ab^	6.67 ± 1.33^a^

*S. flexneri*	SFNR 518	6.67 ± 1.33^a^	6.67 ± 1.33^a^	6.67 ± 1.33^a^	3.33 ± 0.67^ab^	—	3.33 ± 0.67^ab^	1 ± 0.00^b^

*E. coli*	ATCC 25922	5.33 ± 1.33^a^	1.67 ± 0.33^b^	2.67 ± 0.67^ab^	—	—	—	1 ± 0.00^b^
	EC RHB	1.33 ± 0.33^a^	3.33 ± 0.67^a^	—	—	—	—	1.67 ± 0.33^a^

*P. aeruginosa*	PA RHB	—	—	—	—	—	—	2 ± 0.00

*Note:* Plant extracts were tested at 1024 μg/mL and the reference antibiotic (TET: tetracycline) at 512 μg/mL. —: MBC/MIC nondetermined. EtOH: ethanol 70% extract. Values are expressed as mean ± SEM (standard error of the mean). Groups with different letters (a–c) are considered statistically different (*p* < 0.05) using Tukey's multiple comparisons test.

Abbreviations: AqE = aqueous extract; MBC = minimum bactericidal concentration; MIC = minimum inhibitory concentration.

^∗^MBC/MIC < 4 (bacteriostatic) and MBC/MIC > 4 (bactericidal).

**Table 6:** Minimum bactericidal concentrations (MBC in μg/mL) of the studied herbals.

Bacteria strains/isolates		MBC (in μg/mL)						
		Clove		Cinnamon		Thyme		Antibiotic
		AqE	EtOH	AqE	EtOH	AqE	EtOH	TET
*S. aureus*	SANR 46003	682.7 ± 171^ab^	341.3 ± 85.33^b^	—	384.0 ± 128^b^	1024 ± 0.00^a^	853.3 ± 171^a^	213.3 ± 42.67^b^
	SA RHB	512 ± 0.00^a^	426.7 ± 85.33^a^	—	—	—	—	426.7 ± 85.33^a^

*S. enteridis*	SENR 13555	682.7 ± 171^ab^	1024 ± 0.00^a^	—	1024 ± 0.00^a^	1024 ± 0.00^a^	341.3 ± 85.33^b^	512 ± 0.00^b^
	SE CPC	682.7 ± 171^a^	682.7 ± 171^a^	853.3 ± 171^a^	—	—	—	26.67 ± 5.33^b^

*S. typhimurium*	ATCC 14028	512 ± 0.00^a^	512 ± 0.00^a^	853.3 ± 171^a^	—	—	—	53.33 ± 10.67^b^
	STM CPC	256 ± 0.00^b^	426.7 ± 85.33^ab^	853.3 ± 171^a^	853.3 ± 171^a^	—	—	6.667 ± 1.33^c^

*S. paratyphi* B	SPT-B CPC	682.7 ± 171^a^	512 ± 0.00^a^	—	—	—	512 ± 0.00^a^	26.67 ± 5.33^b^

*S. typhi*	ST CPC	682.7 ± 171^ab^	853.3 ± 171^a^	—	—	—	—	213.3 ± 42.67^b^
	ST RHB	—	—	—	—	—	—	—

*S. dysenteriae*	SD CPC	384.0 ± 128^ab^	426.7 ± 85.33^ab^	853.3 ± 171^a^	128 ± 0.00^b^	853.3 ± 171^a^	512 ± 0.00^ab^	26.67 ± 5.33^c^

*S. flexneri*	SFNR 518	128 ± 0.00^b^	96.00 ± 32^b^	512 ± 0.00^a^	853.3 ± 171^a^	—	1024 ± 0.00^a^	128 ± 0.00^b^

*E. coli*	ATCC 25922	682.7 ± 171^ab^	853.3 ± 171^a^	512 ± 0.00^ab^	—	—	—	170.7 ± 42.67^b^
	EC RHB	341.3 ± 85.33^a^	384 ± 128^a^	—	—	—	—	426.7 ± 85.33^a^

*P. aeruginosa*	PA RHB	—	—	—	—	—	—	512 ± 0.00

*Note:* Plant extracts were tested at 1024 μg/mL and the reference antibiotic (TET: tetracycline) at 512 μg/mL. —: MBC > 1024 μg/mL for test herbals and > 512 for TET. EtOH: ethanol 70% extract. Values are expressed as mean ± SEM (standard error of the mean). Groups with different letters (a–c) are considered statistically different (*p* < 0.05) using Tukey's multiple comparisons test.

Abbreviations: AqE = aqueous extract; MBC = minimum bactericidal concentration.

**Table 7:** Types and effects of the interactions of clove extracts (aqueous and hydroethanolic) on the MICs of tetracycline, ciprofloxacin, and erythromycin against the most resistant clinical isolates.

Bacteria isolates	Extracts + antibiotics	MIC (μg/mL)		MIC fold change (ATB)^∗^	FICextract	FICatb	FICi	Outcome
		Alone	Combination					
*E. coli* EC RHB	AqE/TET	341.3 ± 85.33/256 ± 0.00	213.3 ± 42.67/13.33 ± 2.67	−21.33 ± 5.33	0.75 ± 0.25	0.052 ± 0.01	0.80 ± 0.25	Additive
	AqE/CIP	341.3 ± 85.33/13.33 ± 2.67	128 ± 0.00/32 ± 0.00	+0.4167 ± 0.083	0.42 ± 0.083	2.67 ± 0.67	3.08 ± 0.71	Indifference
	AqE/ERY	341.3 ± 85.33/256 ± 0.00	85.33 ± 21.33/32 ± 0.00	−8.00 ± 0.00	0.29 ± 0.11	0.125 ± 0.00	0.42 ± 0.110	Synergy
	EtOH/TET	106.7 ± 21.33/256 ± 0.00	64 ± 0.00/1.67 ± 0.33	−170.7 ± 42.67	0.67 ± 0.17	0.0065 ± 0.001	0.67 ± 0.17	Synergy
	EtOH/CIP	106.7 ± 21.33/13.33 ± 2.67	85.33 ± 21.33/8 ± 0.00	−1.667 ± 0.33	1.00 ± 0.5	0.67 ± 0.17	1.67 ± 0.67	Additive
	EtOH/ERY	106.7 ± 21.33/256 ± 0.00	32.00 ± 0.00/4 ± 0.00	−64.00 ± 0.00	0.33 ± 0.083	0.016 ± 0.00	0.35 ± 0.08	Synergy

*S. typhi* ST RHB	AqE/TET	853.3 ± 171/426.7 ± 85.33	426.7 ± 85.33/4 ± 0.00	−106.7 ± 21.33	0.583 ± 0.22	0.01 ± 0.003	0.59 ± 0.22	Synergy
	AqE/CIP	853.3 ± 170.7/6.67 ± 1.333	256 ± 0.00/13.33 ± 2.67	+0.58 ± 0.22	0.33 ± 0.083	2.33 ± 0.88	2.67 ± 0.96	Indifference
	AqE/ERY	853.3 ± 171/256 ± 0.00	256 ± 0.00/32 ± 0.00	−8.00 ± 0.00	0.33 ± 0.083	0.125 ± 0.00	0.46 ± 0.083	Synergy
	EtOH/TET	1024 ± 0.00/426.7 ± 85.33	213.3 ± 42.67/8 ± 0.00	−42.67 ± 10.67	0.21 ± 0.042	0.026 ± 0.005	0.23 ± 0.039	Synergy
	EtOH/CIP	1024 ± 0.00/6.67 ± 1.33	42.67 ± 10.67/13.33 ± 2.67	1.33 ± 0.33	0.042 ± 0.01	0.8333 ± 0.1667	0.875 ± 0.16	Indifference
	EtOH/ERY	1024 ± 0.00/426.7 ± 85.33	85.33 ± 21.33/64 ± 0.00	−4±0.00	0.083 ± 0.021	0.25 ± 0.00	0.33 ± 0.021	Synergy

*S. aureus* SA RHB	AqE/TET	128 ± 0.00/53.33 ± 10.67	42.67 ± 10.67/0.125 ± 0.00	−426.7 ± 85.33	0.33 ± 0.083	0.002 ± 0.0006	0.34 ± 0.083	Synergy
	AqE/CIP	128 ± 0.00/10.67 ± 2.67	426.7 ± 85.33/8 ± 0.00	1.33 ± 0.33	3.33 ± 0.67	0.83 ± 0.17	4.17 ± 0.83	Antagonism
	AqE/ERY	128 ± 0.00/213.3 ± 42.67	32 ± 0.00/16 ± 0.00	−13.33 ± 2.67	0.25 ± 0.00	0.083 ± 0.021	0.33 ± 0.021	Synergy
	EtOH/TET	106.7 ± 21.33/53.33 ± 10.67	42.67 ± 10.67/16 ± 0.00	−3.33 ± 0.67	0.42 ± 0.083	0.33 ± 0.083	0.75 ± 0.14	Additive
	EtOH/CIP	106.7 ± 21.33/10.67 ± 2.67	32 ± 0.00/16 ± 0.00	1.33 ± 0.33	0.33 ± 0.083	0.83 ± 0.17	1.17 ± 0.22	Indifference
	EtOH/ERY	106.7 ± 21.33/213.3 ± 42.67	64 ± 0.00/53.33 ± 10.67	−5.33 ± 1.33	0.67 ± 0.17	0.21 ± 0.042	0.88 ± 0.13	Additive

*P. aeruginosa* PA RHB	AqE/TET	853.3 ± 171/341.3 ± 85.33	213.3 ± 42.67/8 ± 0.00	−42.67 ± 10.67	0.292 ± 0.11	0.0264 ± 0.005	0.32 ± 0.11	Synergy
	AqE/CIP	853.3 ± 171/21.33 ± 5.33	853.3 ± 170.7/13.33 ± 2.67	1.667 ± 0.33	1.12 ± 0.44	0.67 ± 0.17	1.83 ± 0.44	Indifference
	AqE/ERY	853.3 ± 171/213.3 ± 42.67	426.7 ± 85.33/64 ± 0.00	−3.33 ± 0.67	0.5 ± 0.00	0.33 ± 0.083	0.83 ± 0.083	Additive
	EtOH/TET	1024 ± 0.00/341.3 ± 85.33	26.67 ± 5.33/16 ± 0.00	−21.33 ± 5.33	0.026 ± 0.005	0.052 ± 0.01	0.078 ± 0.016	Synergy
	EtOH/CIP	1024 ± 0.00/21.33 ± 5.33	26.67 ± 5.33/21.33 ± 5.33	0.75 ± 0.25	0.026 ± 0.005	2.00 ± 1.00	2.026 ± 0.99	Indifference
	EtOH/ERY	1024 ± 0.00/341.3 ± 85.33	64 ± 0.00/64 ± 0.00	−4.00 ± 0.00	0.063 ± 0.00	0.25 ± 0.00	0.31 ± 0.00	Synergy

*Note:* EtOH: ethanol 70%. ATB: antibiotic. TET: tetracycline. CIP: ciprofloxacin. ERY: erythromycin. (FICi ≤ 0.5: synergy; 0.5 < FICi ≤ 1: additive; 1 < FICi ≤ 4: indifference; FICi > 4: antagonism). Values are expressed as mean ± SEM (standard error of the mean).

Abbreviations: AqE = aqueous extract; FIC = fractional inhibitory concentration; FICi = fractional inhibitory concentration index; MIC = minimum inhibitory concentration.

^∗^MIC fold change of ATB (+: increase in MIC of antibiotic; −: decrease in MIC of antibiotic).

## Data Availability

The data that support the findings of this study are available from the corresponding authors upon reasonable request.
